# Development of a symptom assessment in patients with myelofibrosis: qualitative study findings

**DOI:** 10.1186/s12955-019-1121-1

**Published:** 2019-04-11

**Authors:** Ruben A. Mesa, Yun Su, Adrien Woolfson, Josef T. Prchal, Kathleen Turnbull, Elias Jabbour, Robyn Scherber, Alan L. Shields, Meaghan Krohe, Funke Ojo, Farrah Pompilus, Joseph C. Cappelleri, Claire Harrison

**Affiliations:** 1 University of Texas Health San Antonio Cancer Care Center, 7979 Wurzbach Rd, San Antonio, TX 78229 USA; 2 Pfizer Inc., 235 E 42nd St., New York, NY 10017 USA; 3 University of Utah School of Medicine, 201 Presidents Cir., Salt Lake City, UT 84112 USA; 4 MD Anderson Cancer Center, 1230 Holcombe Blvd., Houston, TX 77030 USA; 5 Mayo Clinic Cancer Center, 5881 E Mayo Blvd., Phoenix, AZ 85054 USA; 6 Adelphi Values, 290 Congress St. 7th Floor, Boston, MA 02210 USA; 7 Pfizer Inc., 445 Eastern Point Road MS 8260-2502, Groton, CT 06340 USA; 8 Guy’s and St. Thomas’ NHS Foundation Trust, St. Thomas Hospital, Westminster Bridge Rd. Lambeth, London, SE1 7EH UK

**Keywords:** Primary myelofibrosis, Patient reported outcome measures, Symptom assessment, Product labeling, Janus kinase inhibitors, Myeloproliferative disorders

## Abstract

**Background:**

The goal of the research reported here was to understand the patient experience of living with myelofibrosis (MF) and establish content validity of the Modified Myeloproliferative Neoplasm Symptom Assessment Diary (MPN-SD).

**Methods:**

Qualitative interviews were performed in patients with MF, including both concept elicitation and cognitive debriefing. Patients with MF were asked to spontaneously report on their signs, symptoms, and impacts of MF, as well as their understanding of the MPN-SD content, and use of the tool on an electronic platform. A supplementary literature review and meetings with MF experts were also performed.

**Results:**

Twenty-three patients with MF participated in qualitative interviews. Signs and symptoms most commonly reported by ruxolitinib-experienced patients (*n* = 16) were: fatigue and/or tiredness (*n* = 16, 100%), shortness of breath (*n* = 11, 69%), pain below the ribs on the left side and/or stomach pain and/or abdominal pain (*n* = 9, 56%), and enlarged spleen (*n* = 9, 56%) and for ruxolitinib-naïve patients (*n* = 7) were: fatigue and/or tiredness (*n* = 6, 86%), pain below the ribs on the left side (*n* = 6, 86%), enlarged spleen (*n* = 4, 57%), full quickly/filling up quickly (*n* = 4, 57%), night sweats and/or general sweats (*n* = 4, 57%), and itching (*n* = 4, 57%). Patients demonstrated that they were able to read, understand, and provide meaningful responses to the MPN-SD. The final version of the MPN-SD includes the 10 most commonly reported concepts from the MF patient interviews.

**Conclusions:**

The findings demonstrate the comprehensiveness of the MPN-SD in assessing MF symptoms in both ruxolitinib-experienced and ruxolitinib-naïve patients, while remaining easy for patients to understand and complete.

## Background

Myelofibrosis (MF) is a malignant clonal disease characterized by progressive marrow failure, splenomegaly, and decreased longevity due to infections, bleeding, and leukemic transformation [1]. MF comprises numerous burdensome symptoms for patients, including fatigue, night sweats, upper left quadrant abdominal pain, bone pain, and, among others, unintentional weight loss. These symptoms, in turn, often have negative impacts on patients’ quality of life; patients with MF frequently complain of night sweats, severe itching, early fullness or satiety, and a variety of pains in their bones and muscles, under their ribs, and in their abdomen, which all contribute to a reduced health related quality of life (HRQoL) [2].

When assessing the full impact of disease and treatment on HRQoL, it is important to consider the patient’s perspective. Indeed, the European Medicines Agency (EMA) [3] and United States (US) Food and Drug Administration (FDA) [4] acknowledge the importance of the patient’s voice in clinical trials by outlining steps for good patient-reported outcome (PRO) instrument development. The only currently FDA-approved drug for first-line treatment of MF is Jakafi™ (ruxolitinib [RUX]) [5]. RUX is indicated for the treatment of patients with intermediate or high-risk MF, including primary MF (PMF), post-polycythemia vera (PPV-MF), and post-essential thrombocythemia (PET-MF), having been shown to reduce spleen volume and improve MF-associated symptoms in those populations.

The modified Myelofibrosis Symptom Assessment Form (MFSAF) v2.0 diary (a symptom assessment PRO tool) was used successfully to obtain PRO label claims from the FDA [6] and EMA in patients naïve to RUX inhibition (RUX-naïve). Fatigue, a hallmark symptom of MF, is missing from MFSAF v2.0, and it is unclear if this tool is appropriate for patients resistant or intolerant to RUX treatment (RUX-experienced). To overcome these issues, this study aimed to develop a new PRO instrument. Specifically, the goal of this study was to understand the patient perspective of living with MF and establish content validity of a new PRO instrument for administration in both RUX-experienced and RUX-naïve MF patients. Data was collected from three sources: patients, experts, and the published literature. The result of this work contributed to the development of the Myeloproliferative Neoplasm Symptom Assessment Diary (MPN-SD), a PRO instrument designed for the purpose for assessing key symptoms among RUX-experienced and RUX-naïve MF patients.

## Methods

This study was conducted in accordance with the International Society for Pharmacoeconomics and Outcomes Research (ISPOR) PRO Good Research Practices Task Force Report: Part 1 [7] and 2 [8] and FDA PRO Guidance [4], which each specify methods for development of a PRO tool.

### Qualitative interviews with MF patients

#### Patient recruitment

**Fig. 1:**
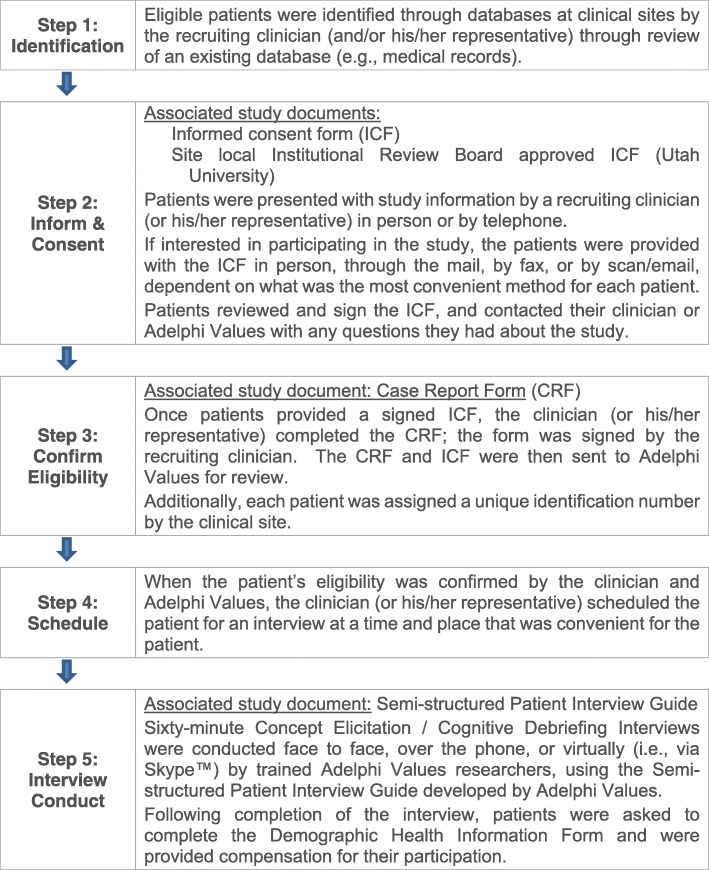
Agency and academic research site patient recruitment process

All study documents were institutional review board-approved prior to study initiation. Interviews were scheduled after a potential participant confirmed his or her interest in participating, signed informed consent and the Health Insurance Portability and Accountability Act (HIPAA) Authorization, and was deemed eligible (see Fig. 1 displaying the recruitment process).

#### Patient eligibility criteria

Eligible patients included those who were ≥ 18 years of age; fluent in US English; willing and able to participate and complete questionnaires; diagnosed with PMF or secondary MF (PET-MF and PPV-MF) as per World Health Organization 2008 criteria; had failed prior treatment with a RUX (RUX-experienced), or had no prior RUX treatment and Intermediate-1, Intermedate-2 or High-risk MF according to the Dynamic International Prognostic Scoring System (RUX-naïve); had a palpable spleen; and had reported current MF symptoms. A patient was not eligible if he or she met any of the following exclusion criteria: previous treatment with a licensed or experimental smoothened inhibitor; targeted anti-cancer therapy up to 14 days prior to enrollment; a history of drug or alcohol abuse within the last 6 months; cognitive impairment; the presence of a psychiatric condition; or any other clinically relevant concern that would interfere with the patient’s ability to provide written informed consent and/or participate.

#### Patient interview methods

A semi-structured interview guide was developed to capture open-ended and probing questions regarding symptoms and impacts of MF from the patients (concept elicitation interviews, CEIs); evaluate the MPN-SD for readability, comprehensibility, relevance, and comprehensiveness (cognitive debriefing interviews, CDIs); and assess the usability of the electronic patient-reported outcome device (ePRO usability testing). CEI, CDI, and ePRO usability testing were conducted during the same interview session. Interviews were audio recorded; conducted face to face, over the telephone, or virtually; and each lasted 60–90 min. Interviewers were trained via the National Institutes of Health Human Participant Protection.

#### Data collection

During CEIs, symptom expressions were tracked; participants ranked the top five symptoms they would like to have improve with treatment; and reported on the impact of MF symptoms. Concepts were tabulated for frequency (i.e., number and percent of spontaneous reports).

For CDIs, the MPN-SD was presented on an ePRO device and patients were asked to “think aloud” about the process they used to arrive at each answer. Data were collected on the words, terms, or concepts that were not understood or were interpreted differently than intended [9]. The MPN-SD was modified mid-way and a second wave of interviews was performed to evaluate the changes. The usability of the ePRO device was evaluated via questionnaire.

Audio-recordings of interviews were transcribed, anonymized, and entered into ATLAS.ti Version 7.0 (ATLAS.ti Scientific Software Development GmbH, Berlin) [10]. A code book was developed based on researchers identifying transcript text relevant to the research objectives and matching it to a code from the coding scheme that best characterized the data [11]. Analysis was performed separately for RUX-experienced and RUX-naïve patient groups. Saturation was evaluated; saturation characterizes the point at which no new information can be generated from additional interviews [7]. The demonstration of saturation is used as evidence for the adequacy of the sample size (if saturation is not observed, additional interviews may be considered).

### Literature review

**Fig. 2:**
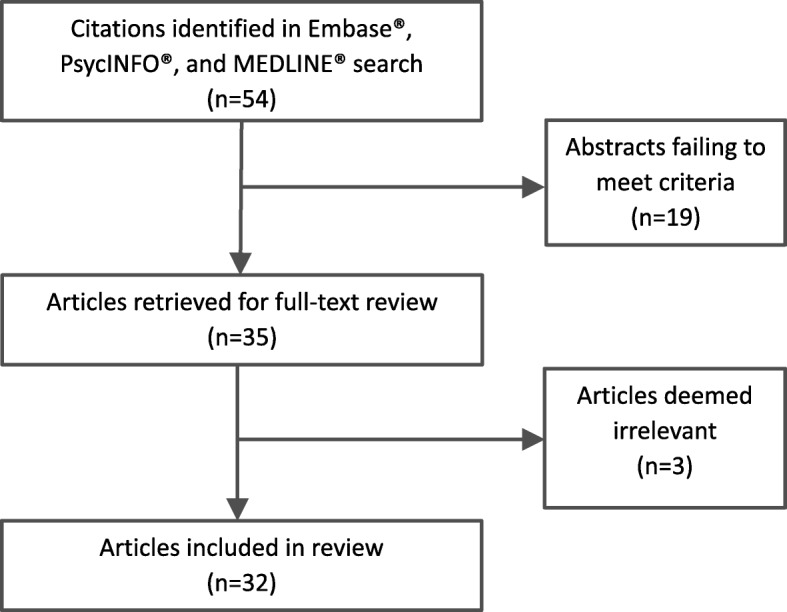
Literature review screening process and article flow

In order to provide further evidence of the relevance and importance of concepts identified during patient interviews, and to help guide revisions to the interview guide between interview waves, a conceptual literature review was conducted concurrent with the start of patient interviews. It was designed to capture key articles that identify, define, and substantiate the symptom-level concepts experienced by adults with MF. The search was conducted January 2015 via the OvidSP platform. Key words included: “myelofibrosis,” “osteomyelofibrosis,” “patient report,” “qol,” “quality,” “signs,” and “symptoms.” The search was limited to humans, US English, and journal publication type. No limits were placed on publication year. Concepts from the literature were categorized and prioritized (reported by ≥5 full text articles) according to symptoms, impacts, or MF treatment (see Fig. 2).

### Expert advice meetings

Interviews with four experts in MF substantiated symptom and impact level concepts of measurement. Experts were identified from a list of clinicians who specialized in hematology. Interviews were conducted over the phone by trained interviewers, followed a semi-structured interview guide, and were each 60 min. The interviews were analyzed using semi-quantitative and qualitative data analytic methods via ATLAS.ti Version 7.0 (ATLAS.ti Scientific Software Development GmbH, Berlin) [10].

## Results

### Qualitative interviews with MF patients

Twenty-three MF patients participated in interviews (RUX-experienced = 16, RUX-naïve = 7). Eleven were recruited and enrolled from Global Market Research Group, three from Mayo Clinic, six from Utah University, and three from the MPN Research Foundation. Interviews occurred between October 2014 and September 2015.

#### Demographics

Mean age for RUX-experienced patients was 67 years (range of 30 to 85, standard deviation [SD] ±15.5). Most were white (*n* = 14, 88%), and male (*n* = 9, 56%). Thirty-one percent of patients reported “fair” general health (*n* = 5) and 69% rated the severity of their condition as moderate (*n* = 11). Mean age for RUX-naïve patients was 59 years (range of 46 to 71, SD ±8.8). All were white/Caucasian (*n* = 7, 100%) and most were male (*n* = 4, 57%). Seventy-one percent of RUX-naïve patients (*n* = 5) reported being in “good” general health and rated the severity of their condition as mild. See Table 1 for additional demographic characteristics.

**Table 1:** Patient reported demographic and health information

	Total combined sample (*N* = 23) N (%)	RUX- experienced total (*n* = 16) n (%)	RUX-naïve total (*n* = 7) n (%)
Gender			
Female	10 (43.5%)	7 (43.8%)	3 (42.9%)
Male	13 (56.5%)	9 (56.3%)	4 (57.1%)
Age			
Range	29.9–85.4	29.9–85.4	45.9–70.7
Mean (SD)	65.5 (12.2)	67.2 (15.5)	58.7 (8.8)
Ethnicity			
Not Hispanic/Latino	23 (100.0%)	16 (100.0%)	7 (100.0%)
Race			
White/Caucasian	21 (91.3%)	14 (87.5%)	7 (100.0%)
Other^a^	2 (8.7%)	2 (12.5%)	0 (0.0%)
Education			
High school diploma (or GED) or less	5 (21.7%)	3 (18.8%)	2 (28.6%)
Some college or certificate program	5 (21.7%)	4 (25.0%)	1 (14.3%)
College or university degree (2- or 4-year)	7 (30.4%)	3 (18.8%)	4 (57.1%)
Graduate degree	6 (26.1%)	6 (37.5%)	0 (0.0%)
Other	1 (4.3%)	1 (6.3%)	0 (0.0%)
Living status			
Living alone	3 (13.0%)	3 (18.8%)	0 (0.0%)
Living with family or friends	18 (78.3%)	12 (75.0%)	6 (85.7%)
Other	2 (8.7%)	1 (6.3%)	1 (14.3%)
Annual household income			
Under $25,000	2 (8.7%)	1 (6.3%)	1 (14.3%)
$25,000 to $49,999	1 (4.3%)	1 (6.3%)	0 (0.0%)
$50,000 to $74,999	6 (26.1%)	4 (25.0%)	2 (28.6%)
$75,000 to $99,999	6 (26.1%)	3 (18.8%)	3 (42.9%)
$100,000 and over	4 (17.4%)	4 (25.0%)	0 (0.0%)
Not answered	4 (17.4%)	3 (18.8%)	1 (14.3%)
Work status			
Working full-time	5 (21.7%)	2 (12.5%)	3 (42.9%)
Working part-time	1 (4.3%)	1 (6.3%)	0 (0.0%)
Retired	14 (60.9%)	12 (75.0%)	2 (28.6%)
Unemployed	1 (4.3%)	0 (0.0%)	1 (14.3%)
On disability	2 (8.7%)	1 (6.3%)	1 (14.3%)
Relationship status			
Single	1 (4.3%)	0 (0.0%)	1 (14.3%)
Have a significant other	18 (78.3%)	13 (81.3%)	5 (71.4%)
Married	1 (4.3%)	0 (0.0%)	1 (14.3%)
Divorced/Separated	2 (8.7%)	2 (12.5%)	0 (0.0%)
Widowed	1 (4.3%)	1 (6.3%)	0 (0.0%)
Health in general			
Excellent	1 (4.3%)	1 (6.3%)	0 (0.0%)
Very good	4 (17.4%)	3 (18.8%)	1 (14.3%)
Good	8 (34.8%)	3 (18.8%)	5 (71.4%)
Fair	6 (26.1%)	5 (31.3%)	1 (14.3%)
Poor	4 (17.4%)	4 (25.0%)	0 (0.0%)
Condition severity			
Mild	6 (26.1%)	1 (6.3%)	5 (71.4%)
Moderate	12 (52.2%)	11 (68.8%)	1 (14.3%)
Severe	5 (21.7%)	4 (25.0%)	1 (14.3%)

^a^1 patient stated “some Native American / northern European”; 1 patient did not specify race

### Concept elicitation interviews

#### Patient-reported symptoms of MF

A total of 29 symptoms were expressed by RUX-experienced patients, and 14 symptoms were reported by RUX-naïve patients. Eleven symptoms were reported by both groups of patients. Eighteen symptoms were reported by RUX-experienced patients only. Three symptoms (appetite loss, cold, and rashes) were reported by RUX-naïve patients only (see Table 2). Table 3 displays examples of MF patient quotes for the prioritized symptom expressions.

**Table 2:** Myelofibrosis primary symptom concepts

Concept	Frequency of RUX-experienced patient reports; *n* = 16 n (%)	Frequency of RUX-naïve patient reports; *n* = 7 n (%)	Total (*N* = 23) N (%)	Concept reported by both RUX-experienced and RUX-naïve patients
Fatigue and/or tiredness	16 (100.0%)	6 (85.7%)	22 (95.7%)	✓
Shortness of breath	11 (68.8%)	0 (0%)	11 (47.8%)	
Pain below the ribs on the left side and/or stomach pain and/or abdominal pain	9 (56.3%)	6 (85.7%)	15 (65.2%)	✓
Enlarged spleen	9 (56.3%)	4 (57.1%)	13 (56.5%)	✓
Uncomfortable abdomen and/or abdominal discomfort and/or bloating and/or feeling full	8 (50.0%)	1 (14.3%)	9 (39.1%)	✓
Full quickly and/or filling up quickly and/or eating less	7 (43.8%)	4 (57.1%)	11 (47.8%)	✓
Night sweats and/or general sweats	6 (37.5%)	4 (57.1%)	10 (43.5%)	✓
Bone pain	6 (37.5%)	1 (14.3%)	7 (30.4%)	✓
Muscle pain and/or muscle cramps	6 (37.5%)	0 (0%)	6 (26.1%)	
Itching	5 (31.3%)	4 (57.1%)	9 (39.1%)	✓
Dizziness	3 (18.8%)	1 (14.3%)	4 (17.4%)	✓
Bruise easily	3 (18.8%)	0 (0%)	3 (13.0%)	
Balance	2 (12.5%)	0 (0%)	2 (8.7%)	
Brain fog	2 (12.5%)	0 (0%)	2 (8.7%)	
Frequent urination	2 (12.5%)	0 (0%)	2 (8.7%)	
Hot flashes	2 (12.5%)	0 (0%)	2 (8.7%)	
Muscle weakness	2 (12.5%)	0 (0%)	2 (8.7%)	
Peripheral neuropathy and/or numbness	2 (12.5%)	0 (0%)	2 (8.7%)	
Weight loss	1 (6.3%)	3 (42.8%)	4 (17.4%)	✓
Appetite loss	0 (0%)	1 (14.3%)	1 (4.3%)	
Cold	0 (0%)	1 (14.3%)	1 (4.3%)	
Joint pain	1 (6.3%)	1 (14.3%)	2 (8.7%)	✓
Rashes	0 (0%)	1 (14.3%)	1 (4.3%)	
Achiness	1 (6.3%)	0 (0%)	1 (4.3%)	
Back pain	1 (6.3%)	0 (0%)	1 (4.3%)	
Burning sensation	1 (6.3%)	0 (0%)	1 (4.3%)	
Fevers and/or chills	1 (6.3%)	0 (0%)	1 (4.3%)	
Gout	1 (6.3%)	0 (0%)	1 (4.3%)	
Hand pain	1 (6.3%)	0 (0%)	1 (4.3%)	
Headaches	1 (6.3%)	0 (0%)	1 (4.3%)	
Inflammation in arm	1 (6.3%)	0 (0%)	1 (4.3%)	
Nausea	1 (6.3%)	0 (0%)	1 (4.3%)

**Table 3:** Example patient expressions

Concept	RUX-experienced example patient expressions	RUX-naïve example patient expressions
Fatigue and/or tiredness	*(05-02-M-74)* *… all of the sudden I started getting more lethargic and, and more tired, more fatigued.* *(01-05-F-55)* *I felt that I was very tired all the time.*	*(01-04-M-50)* *… when I was first diagnosed I’d get really fatigued during the day. Come home, take a three-four hour nap and, uh, never felt any different. Still fatigued …* *(01-14-F-67)* *I was just tired.… But every day around three o’clock I laid down and went to bed and I couldn’t sit up very long.… my body just felt very uncomfortable.*
Shortness of breath	*(01-02-M-73)* *I can’t, sometimes, ah, I feel like I can’t even catch my breath. It’s, it’s getting worse. It’s not getting better at all.…*	N/A
Pain below the ribs on the left side and/or stomach pain and/or abdominal pain	*(03-09-F-29)* *I used to have a lot of spleen pain before I was on treatment.… … it’s upper left … right under where my boob is … all the way to the left. Like on the side.*	*(01-17-M-70)* *… it is definitely on the, uh, left side of the ribcage.… It’s continual.… But it’s not chronic in that the only time I’d really know I have it is if I press on it or I tighten my belt too tight. It’s not that it’s a throbbing continual pain.* *(01-17-M-70)* *… it is definitely on the, uh, left side of the ribcage.… It’s continual.… But it’s not chronic in that the only time I’d really know I have it is if I press on it or I tighten my belt too tight. It’s not that it’s a throbbing continual pain.*
Enlarged spleen	*(03-01-M-66)* *A: Uh, yes.… The spleen pain was very – I knew when that* *pain – I call it abdominal pain because my spleen was so big. It was bigger than a football and, and it went from one side of my body to the other. So whenever it hurt it would hurt in my entire abdomen area, but it was … definitely caused by the spleen. I, I could tell.*	*(01-16-F-59)* *I have, uh, an enlarged spleen, which I can feel.… Well I don’t feel it here when I’m sitting up, but when I lie down* *(01-01-F-58)* *I felt that my spleen might be enlarged, but of course I’m not sure. I couldn’t – I don’t know … So when the spleen becomes enlarged sometimes it feels uncomfortable or you get some sort of pain down there*
Uncomfortable abdomen and/or abdominal discomfort and/or bloating and/or feeling full	*(05-01-F-69)* *Back in December of 2007 … I had been experiencing since the previous October a lot of pain on my left side … feeling very full all the time, very, very kind of bloated and uncomfortable.*	*(01-04-M-50-P)* *I get an almost a nausea feeling from it. Uh, and other than that it means you feel full.*
Full quickly and/or filling up quickly and/or eating less	*(05-01-F-69)* *… feeling very full all the time …* *03-09-F-29)* *I had been noticing, up to that point, I’d been eating less.…I was having a really hard time eating … regular sized meals …*	*(01-04-M-50-P)* *I do have that too.… the feeling of fullness is I would eat – like right now I would eat maybe a quarter of the portion I’m normally used to eating and it just – I can’t eat no more. I’m just full.*
Night sweats and/or general sweats	*(05-03-M-79)* *I was having terrible night sweats.… They were very, very bad sweats.… It’s almost like I was waddling on water and as soon as I went to bed, they started. And then they’d go on all night.… Every night.*	*(01-01-F-58-P)* *Well I wake up and I just feel like a flush of warmth like, um, kind of like hot flashes* *(01-14-F-67)* *…I just can sometimes just sit there and have company and have 10 napkins besides me and just keep, um, just keep trying to wipe away the sweat.*
Bone pain	*(03-07-F-64)* *I would get a lot of long bone pain … particularly when I was lying on my couch.… I’ve only had it in my legs. I’ve never had it substantially in my arms, but … I still will get that in my legs.*	*(01-01-F-58)* *I have bone pain.…* *(01-01-F-58)* *… every time I lie down it hurts for a little while. Um, when I get up it hurts for a little while and if I am say driving for a long time it hurts.*
Muscle pain and/or muscle cramps	*(03-09-F-29)* *… within the first year a few more symptoms kind of started showing up, like … more bone pain, joint pain and muscle pain.*	N/A
Itching	*(01-02-M-73-P)* *...it was the worse thing you ever want to see. I mean, I was slapping myself, it was so bad.… I didn’t want to scratch it because I was leaving … red blotches, so I was slapping myself.*	*(01-01-F-58)* *I had no rash, nothing coming up on my body, but just a very severe itching, particularly after showers.* *(01-01-F-58)* *… in 2013, last year, uh, I had been feeling very severe itching,*
Dizziness	*(01-05-F-55)* *… a little dizzy.*	*(01-04-M-50)* *… weight loss and dizziness.… Uh, it’s like, uh, getting on like one of them kids’ toys and getting spun around and then standing up and you just – it’s – sometimes it’s difficult to stand up. Usually I got to lean up against something for about 10 s for it to go away.*
Bruise easily	*05-01-F-69)* *Well very easily bruising of course because of the lower platelet count.… if I get a cut or, you know, something like that it bleeds for quite a while.… I always, you know, of course have the bruising if I bump into anything.…*	N/A
Balance	*(01-09-F-73)* *The balance is when I get up and I tend to sometimes go to my left and hopefully there’s something there to grab on to. I’m actually considering a cane that – I feel I’m at that point where I should have something to … rely on.… You know, and I’ve learned to get up slowly and …*	N/A
Brain fog	*(03-09-F-29)* *It wasn’t that bad around the time that I was first diagnosed, then even … the first year or so … I didn’t really have it that bad. And I don’t know how much of it is because of the disease or it’s the Jakafi.… I don’t know which one … causes that. Or if it’s just the lack of sleep that I get.… Or … the fatigue …*	N/A
Frequent urination	*(01-09-F-73)* *It’s just … I have to go to the bathroom and when I have to go I better move quickly. Yeah … and it’s more so now because my bladder is being squished more … which I hate. I wear … the little panty liners when I came when I’m home. If I go out I wear a very thick one. And I’ve been looking at … those stupid diapers for adults, which I’m not looking forward to but … just to be on the safe side. ‘Cause if I have to go it’s going. It’s not gonna stop.*	N/A
Hot flashes	*(05-01-F-69)* *I’ve had somewhat of the … night sweats.… I think the only thing I experience at night has more to do with hot flashes than actual night sweats.*	N/A
Muscle weakness	*(01-09-F-73)* *… the legs not wanting to function.… And muscle weakness. I have no muscle whatsoever in my body.… Just no strength.*	N/A
Peripheral neuropathy and/or numbness	*(05-01-F-69)* *I do have some … peripheral neuropathy in my … lower legs … in my shin area and my feet.*	N/A
Weight loss	*(05-03-M-79)* *I went to my family doctor because I had lost a lot of weight.… I could barely stand up.… I found out the reason was that my blood count was so low … Of course I lost so much weight, I lost about 40 pounds in a month*	*(01-11-M-69)* *I just remember that I just started losing weight for no, no apparent reason.… I was losing weight and I didn’t really need to.*
Appetite loss	N/A	*(01-01-F-58)* *I used to eat, uh, more food in, in a meal and I used to have more meals and now I, I often, uh, skip a meal often and, uh, I, uh, I don’t have the appetite, uh, that I used to have.*
Cold	N/A	*(01-03-M-59)* *I notice I get a cold every year, uh, but I don’t know if that’s just [State] in general, you know. When I came back – I never got a cold when I was in [Country]. I got the flu, but not a cold every year.*
Joint pain	*(03-09-F-29)* *I would say within the first year a few more symptoms … started showing up, like … joint pain …*	*(01-11-M-69)* *I usually experience some type of joint pain.…*
Rashes	N/A	*(01-03-M-59)* *So, uh, besides that I get rashes.*
Achiness	*(05-05-M-85)* *I was hurting, aching. … they took me to the doctor to find out why I was weak and lightheaded – and aching.…*	N/A
Back pain	*(01-02-M-73)* *In my back. I have severe back pains since all this has come out, too … I’ve always had back pain but not, nothing like this here.… it would come and go but this one here has come and it stays. It doesn’t leave me. It’s constant.*	N/A
Burning sensation	*(05-01-F-69)* *… felt like you had kind of hot blood running through your … body, especially through your upper torso to where it was a burning sensation.…*	N/A
Fevers and/or chills	*(04-03-M-73)* *Once in a while when I have to go to bed early I need a couple Tylenols I get a fever or a chill, variable between those two extremes. That’s not very common … maybe once every two weeks or so, when I overexert.*	N/A
Gout	*(05-04-F-76)* *… it came on with the myelofibrosis … at one point they started me on the RUX2, and then they said, oh, we forgot to give you the gout medicine, so.… Then they gave me one, and that didn’t hold it, so they gave me a second one. So I was taking it twice a day. I’m only taking it once a day now.…*	N/A
Hand pain	*(03-07-F-64)* *It’s only in my hands. The first time it happened was before I was diagnosed with myelofibrosis … I was in my kitchen up north, walking across the room and got intense pain in the joint, the inner joint, the palm side of one finger, intense, 10. And I looked at it, and it was purple, like I called it a splinter hemorrhage which it’s not correct. It was like a star.… it was like a very specific black and blue point …*	N/A
Headaches	*(01-09-F-73)* *Terrible headaches.*	N/A
Inflammation in arm	*(01-09-F-73)* *It was a lot of fluid in my elbow and it’s, it’s come down here and I’m not quite sure what’s going on there.… it’s very red and sore.… It’s swollen …*	N/A
Nausea	*(05-04-F-76-P)* *I just don’t want to eat. It, it makes me feel sick to my stomach.… Makes me feel like I might vomit.* *(05-04-F-76-P)* *Oh, lots.… every time I pull the food out.… And start – and start eating it. Usually I can get a couple bites before it hits.*	N/A

All 16 (100%) RUX-experienced patients reported fatigue and/or tiredness (“*…all of a sudden I started getting more lethargic and more tired, more fatigued*” [05–02-M-74]), while 11 (69%) reported shortness of breath (“…*I feel like I can’t even catch my breath. It’s, it’s getting worse. It’s not getting better at all.…*” [01–02-M-73]), and nine (56%) reported pain below the ribs on the left side and/or stomach pain and/or abdominal pain (“*I used to have a lot of spleen pain before I was on treatment.…it’s upper left … right under where my boob is … all the way to the left. Like on the side.*” [03–09-F-29]), and enlarged spleen. The signs and symptoms most commonly reported by the RUX-naïve patients were fatigue and/or tiredness (reported by six patients, 86%; “*I’d get really fatigued during the day. Come home, take a three-four-hour nap and, uh, never felt any different. Still fatigued* …” [01–04-M-50]), pain below the ribs on the left side (reported by six patients, 86%; *“…it is definitely on the, uh, left side of the ribcage.… It’s continual.…”* [01–17-M-70]), and enlarged spleen, full quickly/filling up quickly, night sweats and/or general sweats, and itching (reported by four patients, 57%, each).

#### Patient-reported impacts of MF

Thirty-two impact concepts were expressed by RUX-experienced patients and 20 by RUX-naïve patients. The most frequently reported impacts for the RUX-experienced patients were impact of daily activities (*n* = 13, 81%), physical impact (*n* = 13, 81%), emotional and/or psychological impact (*n* = 11, 69%), social impact (*n* = 9, 56%), and impact on work and/or school (*n* = 8, 50%). The most commonly reported impacts for the RUX-naïve patients were social (*n* = 5, 71%), emotional and/or psychological (*n* = 4, 57%), and impact on work (*n* = 4, 57%).

#### Saturation

Concept saturation was achieved for both groups (see Table 4 for analysis of RUX-experienced patients, and Table 5 for analysis of RUX-naïve patients). Based on these saturation results, it was determined that an adequate number of interviews were performed.

**Table 4:** Saturation grid for subjects with MF who are treatment-experienced (*n* = 16)

Concepts	First four interviews vs. next four^a^	First eight interviews vs. next four^a^	First twelve interviews vs. next four^a^	Total^b^	Saturation achieved^c^
Fatigue and/or tiredness	3 vs. 4	7 vs. 3	10 vs. 3	13	Yes
Enlarged spleen	1 vs. 2	3 vs. 2	5 vs. 3	8	Yes
Pain below the ribs on the left side and/or stomach pain and/or abdominal pain	1 vs. 2	3 vs. 0	3 vs. 3	6	Yes
Shortness of breath	4 vs. 0	4 vs. 1	5 vs. 1	6	Yes
Muscle pain and/or muscle cramps	1 vs. 2	3 vs. 0	3 vs. 2	5	Yes
Bone pain	0 vs. 1	1 vs. 1	2 vs. 2	4	Yes
Itching	1 vs. 1	2 vs. 1	3 vs. 1	4	Yes
Night sweats/general sweats	1 vs. 2	3 vs. 1	4 vs. 0	4	Yes
Uncomfortable abdomen and/or abdominal discomfort and/or bloating and/or feeling full	0 vs. 2	2 vs. 0	2 vs. 2	4	Yes
Bruise easily	1 vs. 1	2 vs. 0	2 vs. 1	3	Yes
Dizziness	2 vs. 0	2 vs. 1	3 vs. 0	3	Yes
Balance	2 vs. 0	2 vs. 0	2 vs. 0	2	Yes
Brain fog	0 vs. 0	0 vs. 0	0 vs. 2	2	Questionable
Frequent urination	1 vs. 0	1 vs. 1	2 vs. 0	2	Yes
Full quickly and/or filling up quickly and/or eating less	0 vs. 1	1 vs. 0	1 vs. 1	2	Yes
Muscle weakness	1 vs. 0	1 vs. 1	2 vs. 0	2	Yes
Peripheral neuropathy	0 vs. 2	2 vs. 0	2 vs. 0	2	Yes
Achiness	0 vs. 0	0 vs. 1	1 vs. 0	1	Yes
Back pain	1 vs. 0	1 vs. 0	1 vs. 0	1	Yes
Burning sensation	0 vs. 1	1 vs. 0	1 vs. 0	1	Yes
Fevers/chills	0 vs. 0	0 vs. 1	1 vs. 0	1	Yes
Gout	0 vs. 0	0 vs. 1	1 vs. 0	1	Yes
Hand pain	0 vs. 0	0 vs. 0	0 vs. 1	1	Questionable
Headaches	0 vs. 0	0 vs. 0	0 vs. 1	1	Questionable
Hot flashes	0 vs. 1	1 vs. 0	1 vs. 0	1	Yes
Inflammation in arm	1 vs. 0	1 vs. 0	1 vs. 0	1	Yes
Joint pain	0 vs. 0	0 vs. 0	0 vs. 1	1	Questionable
Weight loss	0 vs. 0	0 vs. 0	0 vs. 1	1	Questionable

^a^Saturation is considered to be reached if a downward trend in the elicitation of new sign and symptom concepts is observed and no new concepts that are relevant (i.e., pertinent to the research question and/or not idiosyncratic to an individual subject) emerge in the final set of interviews. Saturation is considered questionable if new concepts emerge in the final quartile of interview. Subjects included in each group of interviews are as follows: Group 1 (01–05, 01–02, 01–09, and 01–07); Group 2 (03–01, 05–01, 05–02, and 04–01); Group 3 (05–04, 05–05, 05–03, and 04–03); and Group 4 (04–02, 05–07, 03–07, and 03–09)

^b^Total number of subjects who reported the concept spontaneously

^c^Questionable: concepts were considered not relevant to the research question and/or were idiosyncratic to the individual patient

**Table 5:** Saturation grid for subjects with MF who are treatment naïve (*n* = 7)

Root Concept	Interviews 1–3 vs. Interviews 4–5 (first three interviews vs. next two)^a^	Interviews 1–5 vs. Interviews 6–7 (first five interviews vs. last two)^a^	Total^b^	Saturation achieved
Fatigue and/or tiredness	2 vs. 2	4 vs. 2	6	Yes
Pain below the ribs on the left side	2 vs. 1	3 vs. 2	5	
Enlarged spleen	2 vs. 1	3 vs. 1	4	
Night sweats/general sweats	1 vs. 2	3 vs. 0	3	
Weight loss	2 vs. 1	3 vs. 0	3	
Itching	1 vs. 0	1 vs. 1	2	
Appetite loss	1 vs. 0	1 vs. 0	1	
Bone pain	1 vs. 0	1 vs. 0	1	
Cold	1 vs. 0	1 vs. 0	1	
Dizziness	1 vs. 0	1 vs. 0	1	
Full quickly/filling up quickly	1 vs. 0	1 vs. 0	1	
Joint pain	0 vs. 1	1 vs. 0	1	
Rashes	1 vs. 0	1 vs. 0	1	

^a^Saturation is considered to be reached if a downward trend in the elicitation of new sign and symptom concepts is observed and no new concepts that are relevant (i.e., pertinent to the research question and/or not idiosyncratic to an individual subject) emerge in the final set of interviews. Saturation is considered questionable if new concepts emerge in the final quartile of interview. Subjects included in each group of interviews are as follows: Group 1 (01–01, 01–03, 01–04); Group 2 (01–11, 01–14); Group 3 (01–16, 01–17)

^b^Total number of subjects who reported the concept spontaneously

### Cognitive debriefing interview and ePRO usability

The final version of the 10-item MPN-SD includes items on filling up quickly, abdominal discomfort, inactivity, itching, night sweats, pain below the ribs on the left side, bone pain, fatigue (tiredness), shortness of breath, and appetite (loss); see Table 6 for the final version of the MPN-SD. Participants interpreted the MPN-SD instructions, items, and response options as the developers intended, and the concepts were relevant to both RUX-experienced and RUX-naïve patients.

**Table 6:** MPN-SD

INSTRUCTION TEXT: The following screens display questions about your myelofibrosis symptoms. Please rate each symptom at its WORST during the PAST 24 h		
1	During the PAST 24 HOURS, at its WORST, how was your … Filling up quickly when you eat (feeling of fullness soon after you begin to eat)	(Absent) 0 1 2 3 4 5 6 7 8 9 10 (Worst Imaginable)
2	During the PAST 24 HOURS, at its WORST, how was your … Abdominal discomfort (feeling uncomfortable, pressure or bloating)	(Absent) 0 1 2 3 4 5 6 7 8 9 10 (Worst Imaginable)
3	During the PAST 24 HOURS, at its WORST, how was your … Inactivity (including work, home and social activities)	(Absent) 0 1 2 3 4 5 6 7 8 9 10 (Worst Imaginable)
4	During the PAST 24 HOURS, at its WORST, how was your … Night sweats (excessive sweating during sleep)	(Absent) 0 1 2 3 4 5 6 7 8 9 10 (Worst Imaginable)
5	During the PAST 24 HOURS, at its WORST, how was your … Itching	(Absent) 0 1 2 3 4 5 6 7 8 9 10 (Worst Imaginable)
6	During the PAST 24 HOURS, at its WORST, how was your … Bone pain (widespread pain, not joint pain or arthritis)	(Absent) 0 1 2 3 4 5 6 7 8 9 10 (Worst Imaginable)
7	During the PAST 24 HOURS, at its WORST, how was your … Pain below the ribs on the left side	(Absent) 0 1 2 3 4 5 6 7 8 9 10 (Worst Imaginable)
8	During the PAST 24 HOURS, at its WORST, how was your … Fatigue (tiredness)	(Absent) 0 1 2 3 4 5 6 7 8 9 10 (Worst Imaginable)
9	During the PAST 24 HOURS, at its WORST, how was your … Shortness of breath	(Absent) 0 1 2 3 4 5 6 7 8 9 10 (Worst Imaginable)
10	During the PAST 24 HOURS, how was your … Appetite	(Normal appetite) 0 1 2 3 4 5 6 7 8 9 10 (Complete loss of appetite)

The MPN-SD instructions were modified based on two waves of interviews. Eight MF patients provided feedback on the original MPN-SD instructions (five RUX-experienced and three RUX-naïve patients), and five MF patients provided feedback on the alternate instructions (one RUX-experienced and four RUX-naïve patients).

The original instructions “During the PAST 24 HOURS, at its WORST, how was each of the following myelofibrosis symptoms? Please tap one number.” were modified to “The following screens display questions about your myelofibrosis symptoms. Please rate each symptom at its WORST during the PAST 24 HOURS.” More patients interpreted the alternate instructions correctly (92% of RUX experienced [*n* = 13] and 100% of RUX-naïve patients [*n* = 7]) as compared to the original instructions (92% of RUX-experienced and 43% of RUX-naïve patients).

“Bone or muscle pain” in the original MPN-SD was modified to “bone pain” based on feedback from five RUX-experienced and seven RUX-naïve patients. RUX-experienced patients (*n* = 5) indicated that bone pain was more relevant to their experience, expressing that (05–03-M-79) “*Yes, I prefer the other one, the bone pain.… I’m not aware of any muscle pain associated with myelofibrosis*.” Further, separate items for fatigue and tiredness in the original MPN-SD were combined into “fatigue (tiredness)” based on feedback from 12 RUX-experienced and seven RUX-naïve patients. Sixty-one percent of the RUX-experienced and 43% of RUX-naïve patients reported that fatigue and tiredness were the same. Patients expressed that (05–02-M-74) “*…all of the sudden I started getting more lethargic and, and more tired, more fatigued*.”

All RUX-experienced patients spontaneously reported shortness as breath as relevant and important to their experience of MF, five patients mentioned it spontaneously and six confirmed its relevance when probed. Experts also prioritized shortness of breath. Based on these two lines of evidence a shortness of breath item was added to the MPN-SD.

An item for ‘appetite’ was added to the MPN-SD. Appetite loss was relevant and important for treatment naïve patients (14%), by experts, and in the literature. The concept did not come up spontaneously in the treatment experienced patient interviews, although weight loss did.

No revisions were made to either the MPN-SD response options or to the ePRO device. The final MPN-SD includes 10 items, with response options ranging from 0 to 10 (0 = absent, 10 = worst imaginable for nine items, and 0 = normal appetite to 10 = complete loss of appetite for one item). It has a 24-h recall period and is administered via ePRO device. The MPN-SD has a total symptom scoring algorithm. See Table 6 for the final version of the MPN-SD.

### Literature review

Thirty-two full text articles were included in the literature review [1, 2, 12–41]. A total of 32 symptoms were identified from the literature (see Table 7). The symptom concepts most frequently attested in the reviewed literature were: night sweats, fatigue, itching, bone pain, weight loss, fever, abdominal discomfort, early satiety, pain under the left ribs, dyspnea, and appetite loss. All of these, with the exception of weight loss and fever (which are clinically assessed), were also included in the symptom concepts assessed in the MPN-SD.

**Table 7:** Concepts identified in the literature review

Concept	Concept description
Night sweats [1, 2, 12, 13, 15, 17–26, 28–32, 34, 35, 37–39, 41] (including nocturnal sweats)	Episodes of nighttime sweating that soak your nightclothes or bedding and are related to some underlying cause
Fatigue [2, 13, 15, 17, 19–22, 24–30, 33–39, 41]	Feeling of tiredness that lasts a long time and doesn’t go away even after rest
Itching [1, 2, 12, 13, 15, 17–22, 24, 26, 28, 38, 39, 41]	Skin tingling or irritation that makes one want to scratch the itchy area
Bone pain [1, 2, 12, 13, 15, 17, 18, 20–22, 24–26, 28, 32, 34, 37, 39, 41] (including bone tenderness)	Aching or other discomfort in one or more bones
Weight loss [2, 12, 13, 15–17, 19, 21, 23–25, 28–30, 34, 37, 38] (Including undesired weight loss, weight loss attributed to gastric ulcer)	Decrease in body weight, when one did not try to lose the weight on their own
Fever [2, 12, 15, 16, 20, 21, 23–25, 28–30, 35, 37, 38]	Body temperature that is higher than normal
Abdominal discomfort/pain [1, 12, 13, 15, 17, 18, 21, 22, 24–26, 28, 34, 37–39, 41]	Pain/discomfort in the area from below one’s chest to groin
Early satiety [1, 12, 13, 15, 17, 18, 22, 24, 28, 33, 34, 39, 41]	Feeling full sooner than normal or after eating less than usual
Pain under left ribs [1, 2, 16, 18, 22, 26, 28, 39, 41] (including spleen pain, left upper quadrant/subcostal pain, occasional pain or discomfort from the enlarged spleen)	Pain or discomfort located under the ribs on the side of the abdomen
Dyspnea [19, 20, 26–28, 34, 41]	Feeling of not getting enough air (shortness of breath)
Appetite loss [19, 20, 26, 27, 34, 39, 41]	Reduced desire to eat
Pain/discomfort [20, 26, 27, 39, 41]	Feeling triggered in the nervous system. It may be sharp or dull, it may come and go, or it may be constant. It may be felt in one area of the body, such as the back, abdomen or chest or may be felt all over
Cough [21, 22, 25, 33, 34]	Reflex that keeps one’s throat and airways clear
Muscle pain [1, 18, 26, 39, 41]	Involves more than one muscle and can involve ligaments, tendons, and fascia, the soft tissues that connect muscles, bones, and organs
Headache [12, 21] (including chronic headache)	Pain in any region of the head
Numbness/tingling [12, 21, 32] (peripheral neuropathy)	Abnormal sensations that can occur anywhere in your body, but are often felt in your fingers, hands, feet, arms, or legs
Constipation [20, 27]	Having three or fewer bowel movements in a week. The stool can be hard and dry and sometimes it is painful to pass
Diarrhea [20, 27]	Having loose, watery stools more than three times in 1 day. One may also have cramps, bloating, nausea and an urgent need to have a bowel movement
Anorexia [20, 37]	Becoming too thin, but one doesn’t eat enough
Dizziness/vertigo [12, 21]	Dizziness is a feeling of being lightheaded or losing your balance. Vertigo is a feeling that the room is spinning
Nausea [20, 27]	An uneasy or unsettled feeling in the stomach together with an urge to vomit
Swelling [20, 25] (including swollen extremities)	Swelling of extremities (arms and legs)
Altered bowels [25]	Altered movement of food through the digestive tract. This could be in the form of diarrhea, constipation or bowel incontinence
Altered consciousness [28]	Altered awareness
Delirium [28]	Sudden severe confusion due to rapid changes in brain function that occur with physical or mental illness
Easy bruising [37]	Bleeding episodes
Palpitations [28] (including compensatory tachycardia)	Feelings or sensations that your heart is pounding or racing. They can be felt in your chest, throat, or neck
Photophobia [28]	Eye discomfort in bright light
Sweats [16]	Sweating is the release of liquid from the body’s sweat glands. Patients may experience excessive sweating due to MF
Abdominal swelling [14]	When the belly area is bigger than usual
Vomiting [20]	Forcing the contents of the stomach up through the esophagus and out of the mouth
Weakness [28]	Reduced strength in one or more muscles

### Expert advice meetings

Four experts in hematology/oncology, hematology, and internal medicine, who all worked academic (teaching) hospitals, participated in the expert advice meetings. Twenty-four symptoms and 31 impacts were identified by experts. All experts reported that patients with MF generally experience the same set of symptoms pre- and post-RUX treatment failure. See Table 8 for the list of symptoms identified by the experts.

**Table 8:** Expert-reported symptoms of myelofibrosis

Symptoms	Description^a^	Experienced pre and/or post ruxolitinib treatment failure	Frequency of expert report (*N* = 4) n (%)^b^
Fatigue	Lack of energy, feeling of weakness and lethargy	Pre	4 (100%)
		Post	4 (100%)
Night sweats	Excessive sweating at night requiring a change of clothing and/or beddings	Pre	4 (100%)
		Post	4 (100%)
Weight loss	Unintentional loss of weight	Pre	4 (100%)
		Post	4 (100%)
Abdominal pain	Pain associated with an enlarged spleen	Pre	4 (100%)
		Post	3 (75%)
Fever	Low grade fever, feelings of hotness that may or may not be accompanied by sweating. A reoccurrence of fever after receiving ruxolitinib treatment could signal treatment failure or a true infection/progression of disease	Pre	3 (75%)
		Post	3 (75%)
Bone pain	Constant pain experienced all over the body. May respond to ruxolitinib treatment but not completely	Pre	3 (75%)
		Post	2 (50%)
Constipation	Bowel movement once every 3 days characterized by hard stools or straining to go to the bathroom. It could be associated with the enlarged spleen (enlarged spleen blocking patients’ bowels). It is not a common MF symptom	Pre	3 (75%)
		Post	2 (50%)
Poor appetite	Lack of feeling of hunger or not wanting to eat. It is typically associated with early satiety and enlarged spleen	Pre	2 (50%)
		Post	2 (50%)
Early satiety	Feeling full fast after eating a meal. It could be associated with the enlarged spleen	Pre	2 (50%)
		Post	2 (50%)
Abdominal discomfort	Associated with the enlarged spleen	Pre	2 (50%)
		Post	1 (25%)
Diarrhea	Loose bowel movements or loose stools.	Pre	2 (50%)
		Post	1 (25%)
Itching	It is also known as pruritus and can occur all over the body or certain parts of the body. It can occur when a patient comes in contact with a trigger (e.g., water)	Pre	2 (50%)
		Post	1 (25%)
Shortness of breath	Needing to catch one’s breath. It can occur at rest or with exertion	Pre	2 (50%)
		Post	1 (25%)
Abdominal fullness	Excess gas build up in the intestines^c^	Pre	1 (25%)
		Post	1 (25%)
Bleeding	Break in blood vessels such as gastrointestinal, mucosal, and hemorrhoidal bleeding, and nose bleeds. It typically occurs externally.	Pre	1 (25%)
		Post	2 (50%)
Easy bruising	Bruising (blue-like appearance) under the skin due to minor trauma	Pre	1 (25%)
		Post	1 (25%)
Lightheadedness	Feeling of dizziness	Pre	1 (25%)
		Post	1 (25%)
Nausea	Feeling an urge to vomit.^c^ It could be as a result of eating specific kinds of food or the enlarged spleen	Pre	1 (25%)
		Post	1 (25%)
Reflux	Leaking of contents (food or liquid) backwards from the stomach into the esophagus^c^	Pre	1 (25%)
Sweating	Release of liquid from the body’s sweat glands. Patients may experience excessive sweating due to MF^c^	Pre	2 (50%)
Headaches	An ache or pain in the head	Pre	1 (25%)
Impotent	Loss of sexual function	Pre	1 (25%)
Vomiting	Forcing the contents of the stomach up through the esophagus and out of the mouth^c^	Pre	1 (25%)

^a^Expert’s description of the symptom except where noted

^b^Number of experts who reported the symptom

^c^Definition retrieved from the US National Library of Medicine

## Discussion

This work demonstrates the comprehensiveness of the MPN-SD in assessing MF symptoms in RUX-experienced and RUX-naïve patients. The MPN-SD is simple and easy for patients to understand and complete and was developed following the US FDA regulatory guidance on PRO instruments. The patient CEI and CDI, expert interviews, literature review, and usability testing have shown that the MPN-SD has robust content validation.

Concepts identified in the CEIs, CDIs, and usability testing indicated that the MPN-SD is suitable for use in both MF patient groups. Of importance is the applicability of the MPN-SD in the clinical setting and ensuring that the instrument captures the real world experience of MF patients. Experts provided support for the use of the MPN-SD to assess many relevant symptoms of MF in both RUX-naïve and RUX-experienced patients.

A recent internet survey study [2] of 1179 patients with myeloproliferative disorders identified fatigue as one of the most frequently reported symptoms (80.7%) and main contributor to poor quality of life. Indeed, findings from this work indicate that fatigue is a relevant component of MF and if it were improved it would greatly benefit patients’ lives. Given that patients talked about both fatigue and tiredness as the same experience, the two concepts were included in one item on the final MPN-SD.

Several limitations of this study should be considered. Only three of the 16 RUX-experienced patients participated in the ePRO usability interviews; this is a very small sample size to draw conclusions from. The study sample is predominately white, which limits the generalizability of the findings to other racial groups. The CEIs and CDIs were combined in this study; one of the main criticisms of this approach is respondent bias. To attempt to reduce bias, the interview order was randomized. The study reported herein was completed in 2015; thus it does not reflect literature published after 2015, and more recent information could be available at the time of this publication.

The next step in development of the MPN-SD is psychometric evaluation. Demonstration of adequate psychometric performance (e.g., score reliability, construct-related validity, and sensitivity to change) and the creation of score interpretation guidelines (e.g., using anchor-based analyses) could facilitate the use of the MPN-SD to support the development, regulatory approval, and expedited availability of novel medicines for MF patients.

## Conclusion

Improved understanding and development of an appropriate measurement tool to assess patients’ experiences of MF expands a perspective that was previously based primarily on tools developed for RUX-naïve patients. This work may help identify critical target areas for evaluation in clinical studies and guide investigators in selecting outcome variables suitable for intervention for MF patients.

## Abbreviations

CDI: Cognitive debriefing interview
CEI: Concept elicitation interview
EMA: European Medicines Agency
ePRO: Electronic patient-reported outcome
FDA: United States Food and Drug Administration
HIPAA: Health Insurance Portability and Accountability Act
HRQoL: Health-related quality of life
ISPOR: International Society for Pharamacoeconomics and Outcomes Research
MF: Myelofibrosis
MFSAF: Myelofibrosis Symptom Assessment Form
MPN-SD: Myeloproliferative Neoplasm Symptom Assessment Diary
PET-MF: Post-essential thrombocythemia myelofibrosis
PMF: Primary myelofibrosis
PPV-MF: Post-polycythemia vera myelofibrosis
PRO: Patient-reported outcome
RUX: Ruxolitinib
US: United States
